# Mapping the landscape of vitamin D in chronic and idiopathic pain: A bibliometric analysis (2000–2023)

**DOI:** 10.1097/MD.0000000000048575

**Published:** 2026-05-08

**Authors:** Zhaohui Jiang, Yijie Wang, Wei Zhang, Zhenzhen Hu, Fang Lin, Zhou Lin

**Affiliations:** a Department of Orthopedics, Wenzhou Central Hospital, Wenzhou, China; b Department of Intensive Care Unit, Wenzhou Central Hospital, Wenzhou, China.

**Keywords:** bibliometric analysis, chronic pain, fibromyalgia, inflammation, pain management, vitamin D

## Abstract

**Background:**

Recent studies have revealed that the anti-inflammatory properties of vitamin D may play a role in pain regulation. To systematically map the global research landscape on vitamin D and chronic or idiopathic pain from 2000 to 2023, this study aims to identify publication trends, influential countries, institutions, authors, and journals, as well as to characterize the evolution of research themes and emerging hotspots using bibliometric methods.

**Methods:**

Using the keywords “vitamin D,” “pain,” “idiopathic pain,” and “fibromyalgia” as search terms, a systematic search was conducted in the Web of Science Core Collection to identify relevant literature published between January 2000 and December 2023. After screening according to predefined inclusion and exclusion criteria, a total of 1899 articles were included. Using VOSviewer and CiteSpace software, we analyzed bibliometric data, including publication volume, citation metrics, Hirsch index, and journal impact factors, and visually presented collaboration networks, co-citation patterns, and keyword analysis results.

**Results:**

A total of 1899 records were included. The number of research papers on vitamin D and chronic and idiopathic pain has steadily increased, rising from 14 in 2000 to a peak of 184 in 2021. The United States leads research output with 500 papers (26.33% of the total), followed by Italy and the United Kingdom. Harvard University and the University of Sydney ranked as the top institutions, demonstrating robust international collaboration. Co-citation analysis revealed distinct thematic clusters. Keyword analysis indicated that inflammation, osteoporosis, and vitamin D receptor genetics were primary research directions, with recent trends focusing on inflammation and vitamin D supplementation.

**Conclusion:**

This bibliometric analysis indicates that academic interest in the relationship between vitamin D and chronic pain is steadily increasing year by year, with developed countries making particularly significant contributions to this field of research. However, further in-depth exploration is required to definitively establish the clinical value of vitamin D in pain management. Meanwhile, the findings also highlight the importance of international collaboration and suggest that vitamin D may hold greater potential in the treatment of chronic pain.

## 1. Introduction

Chronic pain is a major global public health issue, defined as discomfort lasting longer than 3 months. Currently, approximately 1.5 billion people worldwide are affected, with prevalence rates ranging from 20% to 31%.^[1–4]^ It not only severely impacts patients’ daily functioning and quality of life but is also frequently accompanied by psychological distress.^[5–8]^ Simultaneously, chronic pain imposes a substantial social and economic burden, costing the United States alone between $560 billion and $635 billion annually in healthcare expenditures and lost productivity.^[9,10]^ Idiopathic pain (referred to as chronic primary pain in the International Classification of Diseases, 11th Revision^[11]^), due to its unknown etiology, presents particular treatment challenges. Existing common therapies have significant limitations: opioid medications carry risks of addiction and side effects, nonsteroidal anti-inflammatorydrugs may cause gastrointestinal and cardiovascular issues,^[12,13]^ and over 30% of patients experience inadequate pain relief.^[14]^ Consequently, there is an urgent need to explore novel treatment approaches based on new mechanisms. Beyond regulating calcium homeostasis, vitamin D has demonstrated novel functions. Its receptors are widely distributed throughout the nervous system, immune cells, and skeletal muscle, enabling it to influence pain signal transmission.^[15–17]^ Key mechanisms include immunomodulation by suppressing pro-inflammatory factors such as tumor necrosis factor-alpha and interleukin-1 beta, upregulating neurotrophic factors such as brain-derived neurotrophic factor and nerve growth factor, and exerting neuroprotective effects through calcium channel regulation.^[18]^ Approximately 15.7% of the global population suffers from vitamin D deficiency, with deficiency rates exceeding 39% among the elderly and populations in high-latitude regions.^[19]^ Epidemiological studies consistently associate vitamin D deficiency with multiple pain syndromes. Serum 25-hydroxyvitamin D (25(OH)D) levels in fibromyalgia patients are lower than in healthy controls,^[20]^ with 93% of patients with refractory chronic musculoskeletal pain exhibiting deficiency.^[21]^ The severity of osteoarthritis also shows a negative correlation with vitamin D levels.^[22]^ However, results from randomized controlled trials (RCTs) are inconsistent. Some studies indicate limited efficacy of vitamin D supplementation in improving pain. These discrepancies may relate to differences in patients’ baseline vitamin D levels, supplementation regimens, and pain types.^[23–26]^

The relationship between vitamin D and pain remains largely unexplored. Researchers have yet to fully elucidate the precise role of vitamin D receptors (VDRs) in pain pathways,^[27]^ nor has consensus been reached on biomarkers for patient stratification or optimal treatment regimens.^[27]^ Clinical evidence also varies across different study populations and intervention designs.^[28–30]^ Despite a steady increase in related research over the past 2 decades, a comprehensive, systematic integration of this field remains lacking. Urgent questions include identifying trends in global research output, shifts in research focus, and the connections between foundational research and emerging directions.

Bibliometric analysis is a quantitative research method that applies mathematical and statistical approaches to scientific publications to evaluate research productivity, collaboration patterns, intellectual structures, and thematic evolution within a specific field. It has been widely used in medicine to map research landscapes in areas such as oncology, orthopedics, pain medicine, and nutritional science, particularly when a field has expanded rapidly, and traditional narrative reviews are insufficient to capture its complexity.^[31,32]^ By visualizing citation networks, co-authorship relationships, and keyword co-occurrence patterns, bibliometrics provides a macroscopic perspective that complements systematic reviews and meta-analyses, without attempting to assess evidence quality or clinical efficacy. This study pioneers the use of bibliometrics to address these gaps. We collected relevant literature from 2000 to 2023, identifying key research contributors and collaborative networks within the field. This analysis traced the evolution of research themes and compared shifts between foundational studies and emerging directions. As the first systematic review of vitamin D in chronic and idiopathic pain, it aims to inform future research, foster interregional collaboration, improve mechanism study design, and advance evidence-based pain management.

## 2. Materials and methods

### 2.1. Data sources and search strategies

The Web of Science (WoS) database is widely recognized as one of the most authoritative bibliometric platforms, offering extensive and rigorous coverage of scholarly publications across disciplines.^[33]^ For this study, we conducted a systematic search for articles containing the keywords “chronic pain,” “idiopathic pain,” and “vitamin D” within the period from 2000 to 2023, using the Web of Science Core Collection (WoSCC). Publications from 2024 onward were not included because citation data for very recent articles are incomplete and unstable, which may bias bibliometric indicators such as citation counts and co-citation networks. Therefore, December 31, 2023, was selected as the cutoff date to ensure data reliability and comparability.

**Figure 1.**
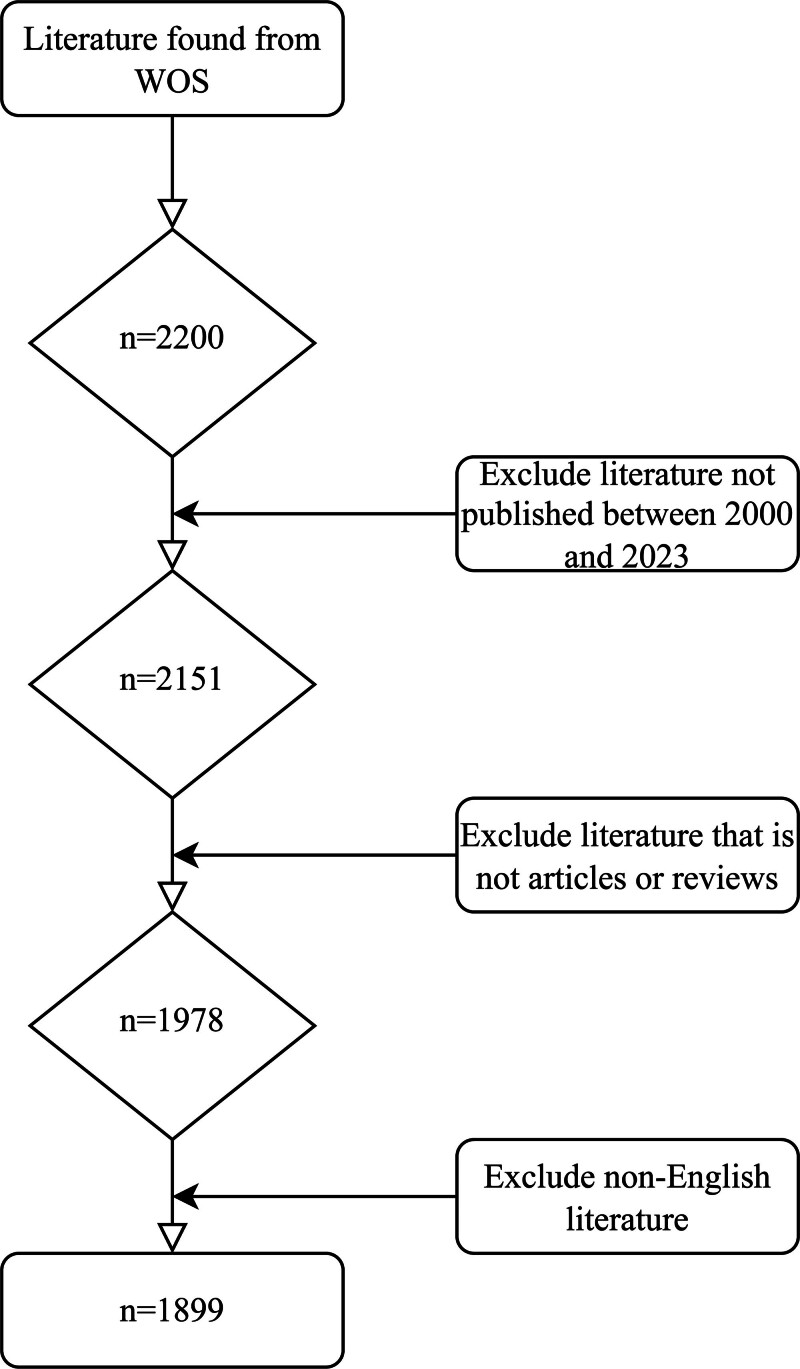
Flow diagram of the literature selection process. WoS = Web of Science.

To ensure consistency and reproducibility, the search was executed on July 1, 2024. The search query was structured as ((((TS=(vitamin D)) OR TS=(cholecalciferol)) OR TS=(ergocalciferol)) OR TS=(calcitriol)) OR TS=(25-hydroxyvitamin D) AND ((TS=(pain)) OR TS=(fibromyalgia)) OR TS=(idiopathic pain), focusing exclusively on peer-reviewed articles and reviews published between January 1, 2000, and December 31, 2023. Only English-language publications were considered. We excluded conference abstracts, editorials, letters, proceedings, corrigenda, news articles, book chapters, case reports, withdrawn papers, reprints, and any documents lacking a WoSCC number. This refined search yielded a total of 1899 articles. Each article was independently assessed by 2 researchers. A detailed outline of the selection methodology is provided in Figure 1. During the eligibility assessment, records were excluded due to nonrelevant topics, non-article publication types, duplication, or lack of complete bibliographic information. Reasons for exclusion at each stage are summarized in the flowchart.

### 2.2. Data management and bibliometric analysis

A comprehensive dataset in “txt” format was obtained from the WoS database, encompassing complete records and citation data for each entry. The dataset includes critical bibliometric information, such as publication details, citation counts, year of publication, institutional affiliations, references, keywords, Hirsch index (H-index) values, countries, regions, author names, and journal titles. In the initial phase of data processing, we focused on merging duplicate entries, rectifying spelling errors, and eliminating irrelevant terms to ensure data accuracy and consistency. Data cleaning procedures included standardizing author names, institutional affiliations, and keywords, merging synonyms, and manually correcting spelling variations to ensure consistency and accuracy prior to analysis.

For the quantitative analysis of bibliometric data, we utilized publication counts and citation metrics – excluding self-citations – as key indicators to evaluate research productivity and impact, respectively. These metrics facilitate a data-driven assessment of research quality. Additionally, the H-index was employed as a metric to gauge scholarly achievement and to potentially forecast future scientific contributions. Journal impact factors (IFs), sourced from the most recent Journal Citation Reports, were also considered an established standard of evaluation.

Data analysis was primarily conducted using Microsoft Excel 2019 (Microsoft Corporation, Redmond) to track annual publication and citation trends. Furthermore, advanced bibliometric and visualization analyses were performed using VOSviewer (version 1.6.18, Leiden University, The Netherlands) and CiteSpace (version 6.2.R2, Drexel University, Philadelphia). Unless otherwise specified, all journal IFs cited refer to the year 2024.

### 2.3. Reporting guideline

This bibliometric study was conducted and reported in accordance with established methodological recommendations for bibliometric and scientometric analyses,^[34,35]^ including the general principles outlined by Donthu et al and Chen et al. In addition, the study design and reporting structure were informed by the Preferred Reporting Items for Systematic Reviews and Meta-Analyses extension for Scoping Reviews framework, where applicable, particularly with respect to literature identification, eligibility assessment, and transparent reporting of the study selection process.^[36]^

### 2.4. Ethical considerations

This study did not involve human subjects or in vivo animal experiments, as it exclusively utilized scientometric data derived from published sources. Consequently, ethical approval was not applicable.

## 3. Results

### 3.1. Publication and citation trends

**Figure 2.**
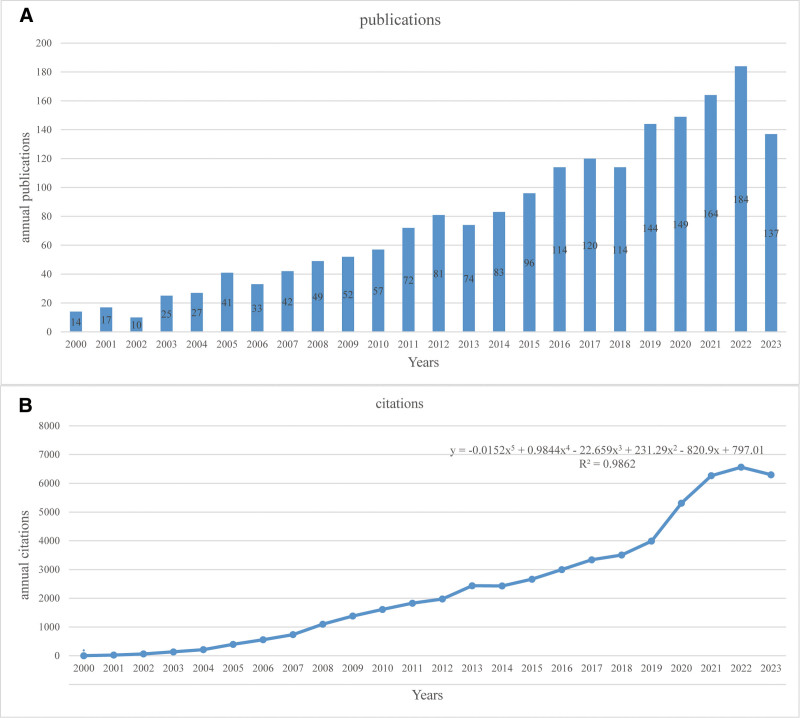
Trends in annual publications and citations from 2000 to 2023. (A) Annual publication trends (2000–2023). This bar graph displays the annual number of publications from 2000 to 2023. The x-axis represents the years, and the y-axis shows the number of publications annually. (B) Annual citation trends (2000–2023). The line graph depicts the annual number of citations received by publications from 2000 to 2023. The x-axis represents the years, and the y-axis indicates the number of citations each year. A polynomial regression line (dotted) is also plotted to show the trend, with the equation and *R*^2^ value provided in the graph.

The temporal analysis of publication data from 2000 to 2023 reveals a consistent upward trend in the number of publications per year (Fig. 2A). In 2000, 14 publications were recorded, followed by a gradual annual increase. Notably, from the mid-2000s, the rate of publication growth accelerated, reaching 72 publications per year by 2011. The publication count peaked at 184 in 2021, after which a slight decline was observed, with 137 publications recorded in 2023.

Similarly, the citation data exhibited a positive trend over the same period (Fig. 2B). Citations remained relatively low, with fewer than 1000 citations per year until 2007. However, from 2008 onward, citation counts began to rise, with marked acceleration from 2014. By 2020, annual citations approached 6000, peaking at approximately 6500 in 2022. A slight stagnation in citations was observed in 2023, suggesting a possible stabilization in citation frequency. A polynomial regression model fitted to the citation data, with an 𝑅^2^ value of 0.9862, demonstrates an excellent fit, indicating that the model captures the underlying trend with high accuracy.

### 3.2. Analysis of research output by countries and institutions

This study assessed the research contributions of the top 10 countries based on a dataset of 1899 records, focusing on the number of publications, percentage of total publications, citation counts, and H-index values. As presented in Table 1, the United States leads with 500 publications, representing 26.33% of the total output. Additionally, the US demonstrates the highest scientific impact, with 22,039 citations and an H-index of 76. Italy ranks second with 187 publications (9.85% of the total), 4950 citations, and an H-index of 37. The United Kingdom follows closely with 179 publications (9.43%), 9345 citations, and an H-index of 51. The People’s Republic of China ranks fourth with 176 publications (9.27%), 2795 citations, and an H-index of 30.

**Table 1:** Top 10 countries in terms of number of articles published on vitamin D research.

Rank	Countries/regions	Np	% of (1899)	Nc	H-index
1	United States	500	26.33	22,039	76
2	Italy	187	9.85	4950	37
3	United Kingdom	179	9.43	9345	51
4	China	176	9.27	2795	30
5	Germany	126	6.64	5360	40
6	Australia	117	6.16	4370	34
7	Turkey	110	5.79	1160	19
8	Japan	83	4.37	2242	20
9	France	77	4.06	3283	29
10	Canada	71	3.74	4557	29

Nc = citation counts, Np = number of publications.

Table 2 lists the top 10 institutions contributing to research in the intersection of pain and vitamin D. Harvard University and Harvard Medical School rank first and second, with 61 and 38 publications, respectively, underscoring North America’s dominant position in global research. The University of London and the University of Oxford rank third and fourth, with 46 and 32 publications, respectively, reflecting Europe’s prominence in higher education and research. Further supporting Europe’s strength in medical research, the French National Institute of Health and Medical Research (INSERM) contributes 41 publications, followed by APHP (38 publications) and the City University of Paris (37 publications). In Oceania, the University of Sydney (45 publications) and the University of Tasmania (31 publications) illustrate the region’s active role in global research networks. The Egyptian Knowledge Bank, with 32 publications, represents Africa’s growing presence in scientific research.

**Table 2:** Top 10 institutions in terms of number of articles issued on vitamin D research.

Rank	Affiliations	Np	% of (1899)	Nc	H-index
1	Harvard University	61	3.212	2677	25
2	University of London	46	2.422	2535	25
3	University of Sydney	45	2.37	2221	18
4	Institut National de la Santé et de la Recherche Médicale (INSERM)	41	2.159	1794	22
5	Assistance Publique–Hôpitaux de Paris (APHP)	38	2.001	1926	23
6	Harvard Medical School	38	2.001	1439	19
7	Université Paris Cité	37	1.948	1869	22
8	Monash University	35	1.843	1129	18
9	Egyptian Knowledge Bank (EKB)	32	1.685	420	12
9	University of Oxford	32	1.685	1945	21
10	University of Tasmania	31	1.632	791	16

Nc = citation counts, Np = number of publications.

The geographic distribution of research output highlights the dominance of developed countries in global scientific infrastructure and funding, while also showcasing the disparities in academic contributions across regions. Notably, although the United States leads in total research output, it only accounts for 2 of the top 10 institutions. China and Japan, despite their national output, lack institutions ranked among the top global research centers, pointing to room for development at the institutional level.

**Figure 3.**
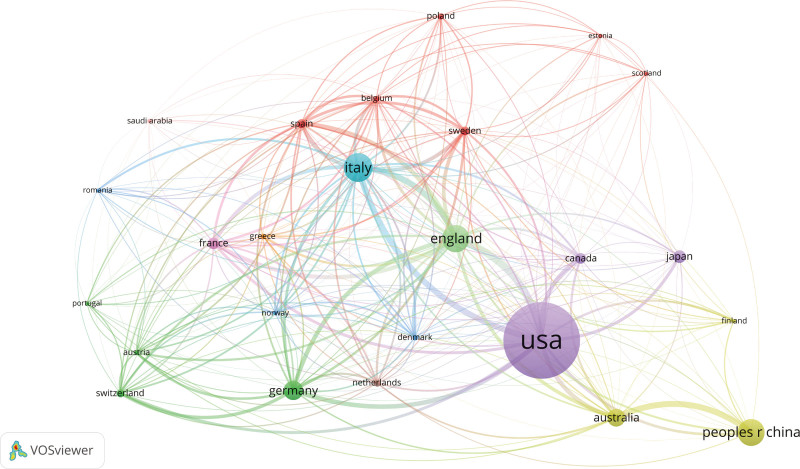
International collaboration network in academic research. This network visualization maps the international collaboration among countries based on academic research data. Each node represents a country, with the node size proportional to the volume of collaborations that the country is involved in. The connections between nodes indicate collaborative relationships, with the line thickness representing the frequency of collaborations and the line color depicting different collaboration clusters.

Network analysis reveals patterns of international academic collaboration. Figure 3 illustrates the global collaboration network at the country level, with the United States occupying the largest node, reflecting its central role in international research partnerships. Other prominent nodes include Italy, the United Kingdom, Germany, and the People’s Republic of China, all of which exhibit extensive collaboration networks. Notably, European countries, particularly Italy, France, Germany, and the United Kingdom, form dense clusters of regional collaboration, reflecting the strength of intra-European research partnerships. The United States also maintains extensive collaborations with countries worldwide, including Canada, the United Kingdom, Germany, and Australia. Meanwhile, collaborations between Asian countries, particularly Japan and China, and Western countries such as the United States and Australia, are on the rise.

**Figure 4.**
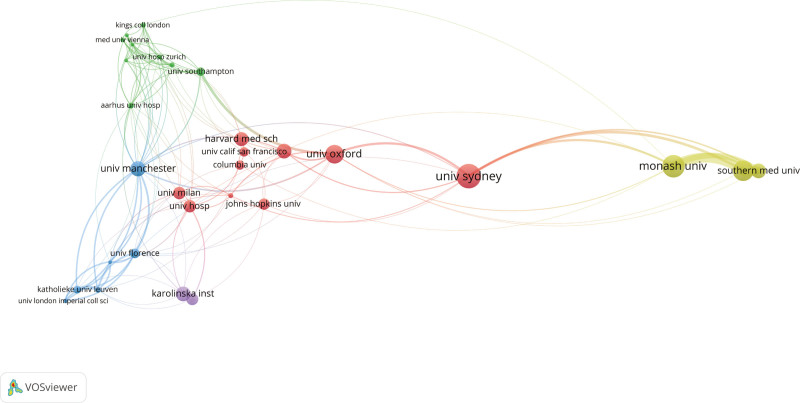
Collaborative network map of institutions involved in the research field. The map visualizes the collaborative relationships between various institutions based on co-authorship data. Larger nodes represent institutions with higher numbers of publications, while the thickness of the connecting lines indicates the strength of collaboration between institutions.

Figure 4 provides insights into collaborative relationships at the institutional level. The University of Sydney, centrally positioned in the network, exhibits strong ties with leading global institutions, such as Oxford University and Harvard Medical School, emphasizing its prominence within the international academic community. European institutions, including King’s College London, the University of Milan, and Karolinska Institutet, are interconnected through numerous collaborations, as indicated by green, blue, and purple lines. These collaborations extend to other regions, highlighting Europe’s active role in global research. North American institutions such as Harvard Medical School, Johns Hopkins University, and Columbia University also demonstrate strong collaborative ties with European counterparts, as evidenced by the blue and green lines. Partnerships between Australian and Asian institutions, such as Monash University and Southern Medical University, are represented by yellow and green lines. These partnerships appear less established, suggesting they may be in early stages of development or ongoing growth. Overall, the network analysis reveals the polycentric nature of global academic collaborations and highlights complementary relationships between regional and cross-regional collaborations.

### 3.3. Analysis of the author’s activities

Table 3 presents the top 10 authors ranked by research output, detailing the number of publications, citations, H-index, and their respective institutional affiliations. Jones G, leading with 20 publications, 826 citations, and an H-index of 14, exemplifies significant academic influence and productivity in this field. Notably, he is affiliated with the University of Tasmania, which features 3 additional authors in the top 10, highlighting the institution’s prominent role in advancing research in this area. Ding CH, affiliated with Southern Medical University, ranks second with 18 publications, 547 citations, and an H-index of 11, reflecting the broad recognition of his scholarly contributions. Antony B and Winzenberg T, both affiliated with the University of Tasmania, published 17 and 16 papers, respectively, achieving notable citation counts, further affirming the institution’s leadership in this research domain. Boonen S, affiliated with KU Leuven, despite contributing only 10 publications, leads all authors with an impressive citation count of 1876, underscoring the profound impact of his research. Overall, the distribution of prolific authors across institutions indicates a concentration of research leadership within specific universities, while the remarkable contributions of individual researchers remain equally noteworthy.

**Table 3:** Top 10 authors in terms of number of articles published on vitamin D research.

Rank	Author	Np	Nc	H-index	Affiliations
1	Jones G	20	826	14	University of Tasmania
2	Ding CH	18	547	11	Southern Medical University
3	Antony B	17	385	9	University of Tasmania
4	Cicuttini F	16	228	8	Monash University
5	Ringe JD	16	549	10	University of Cologne
6	Winzenberg T	16	431	10	University of Tasmania
7	Arden NK	11	461	9	University of Oxford
8	Blizzard L	10	331	8	University of Tasmania
9	Boonen S	10	1876	10	KU Leuven
10	Brandi ML	10	533	9	Stabilimento Chim Farmaceut Mil Firenze
10	Schacht E	10	158	7	University of Cologne

Nc = citation counts, Np = number of publications.

### 3.4. Analysis of co-citations and co-cited references

Co-citation analysis, utilizing bibliometric tools such as VOSviewer, is a robust method for identifying latent connections between scientific publications. Co-citation occurs when 2 documents are cited together by a third source, and frequent co-citation typically indicates a thematic or conceptual relationship between the documents.

**Figure 5.**
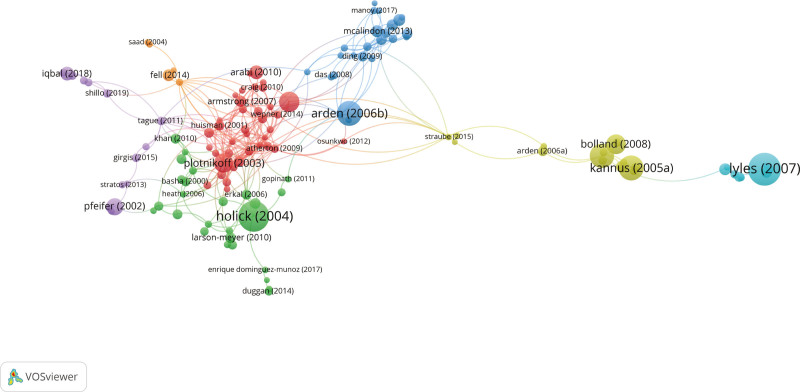
Co-citation analysis network of influential references. The network visualizes the relationships between frequently co-cited references, where each node represents a cited paper, and the size of the node reflects the frequency of citations. The lines between nodes represent co-citation links, with the thickness indicating the strength of co-citation frequency.

Figure 5 presents the co-citation analysis results as color-coded clusters, each representing a distinct research domain. The green cluster, anchored by Holick (2004), and the red cluster, centered on Plotnikoff (2003), exhibit strong interconnections, implying that these bodies of literature explore overlapping themes, particularly the mechanisms of vitamin D action and its health implications. The blue cluster, centered on Arden (2006b), appears to focus on osteoarthritis and other chronic diseases. The structure of the co-citation network in this cluster highlights significant findings and methodological innovations within these areas of research.

The green cluster, anchored by Pfeifer (2002), and the purple cluster, centered on Holick (2004), likely pertain to biochemical or physiological investigations, emphasizing a more microscopic level of analysis. In contrast, the yellow cluster, focused on Bolland (2008) and Kannus (2005a), appears to represent literature addressing specific case studies or particular therapeutic interventions, as indicated by its relative isolation within the co-citation network.

**Figure 6.**
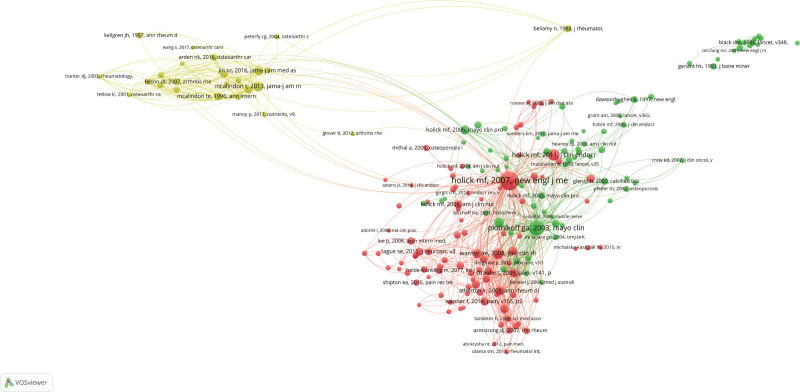
Co-citation network of references in the research field. Each node represents a reference, and the size of the node reflects the frequency of co-citations. The edges between nodes indicate co-citation relationships, where the thickness of the edge represents the strength of the co-citation link. Clusters of frequently co-cited references are color-coded, with prominent clusters centered around key references.

The co-citation network depicted in Figure 6 further elucidates the relationships among distinct research themes. The red cluster, anchored by Holick MF’s 2007 publication in the *New England Journal of Medicine*, is characterized by numerous publications and dense interconnections, indicating that vitamin D and its associated health concerns represent a mature and well-established area of research. The yellow cluster, centered on McAlindon T’s 2013 publication in *JAMA*, appears to focus on osteoarthritis and other chronic conditions. This cluster exhibits fewer co-citation relationships compared to the red cluster, suggesting a more decentralized research landscape or the presence of several parallel research trajectories within this domain.

The green cluster, anchored by Black DM’s 1996 publication in *The Lancet*, appears to focus on osteoporosis and bone health. This cluster exhibits sparse connections to other clusters, reflecting the specificity and relative independence of osteoporosis research within the broader field. Additionally, literature on the periphery of the network, such as Grant AM (2006) and Crew KD (2004), may indicate emerging research directions or specialized investigations into specific issues within the field.

### 3.5. Article and journal analysis

Table 4 presents the ten most-cited articles on “vitamin D” or “cholecalciferol” in relation to “pain” or “fibromyalgia.” The most-cited article, authored by Kenneth W. Lyles and published in 2007 in the *New England Journal of Medicine*, focused on the use of zoledronic acid in reducing clinical fractures and mortality after hip fracture, amassing 1352 citations. Michael F. Holick’s 2004 article in the *American Journal of Clinical Nutrition*, which highlighted the role of vitamin D in preventing various diseases, garnered 1202 citations. Articles from other high-impact journals, including *The Lancet* and *BMJ*, also figure prominently, underscoring the substantial contributions to this field.

**Table 4:** Top 10 most-cited papers in vitamin D research.

Rank	Article title	Year	Journal	Impact factor	Total citations	First author
1	Zoledronic acid and clinical fractures and mortality after hip fracture	2007	New England Journal of Medicine	96.2	1352	Kenneth W Lyles
2	Vitamin D: importance in the prevention of cancers, type 1 diabetes, heart disease, and osteoporosis	2004	The American Journal of Clinical Nutrition	6.5	1202	Michael F Holick
3	Degeneration of the intervertebral disc	2003	Arthritis Research & Therapy	4.4	916	Jill PG Urban
4	Prevention of falls and consequent injuries in elderly people	2005	The Lancet	98.4	784	Pekka Kannus
5	Osteoarthritis: epidemiology	2006	Best Practice & Research Clinical Rheumatology	4.5	778	Nigel Arden
6	Interventions for preventing falls in older people living in the community	2012	Cochrane Database of Systematic Reviews	8.8	757	Lesley D Gillespie
7	Vitamin D insufficiency	2011	Mayo Clinic Proceedings	6.9	546	Tom Thacher
8	Vascular events in healthy older women receiving calcium supplementation: randomised controlled trial	2008	BMJ	93.6	479	Mark J Bolland
9	Prevalence of severe hypovitaminosis D in patients with persistent, nonspecific musculoskeletal pain	2003	Mayo Clinic Proceedings	6.9	462	Gregory A Plotnikoff
10	Effects of the calcimimetic cinacalcet HCl on cardiovascular disease, fracture, and health-related quality of life in secondary hyperparathyroidism	2005	Kidney International	14.8	377	John Cunningham

Table 5 lists the top ten journals by number of publications. *Nutrients* leads with 50 articles, an IF of 4.8, and an average of 21.48 citations per article. *Osteoporosis International* ranks second with 38 articles, while demonstrating a higher average number of citations per article at 54.42. *The Journal of Bone and Mineral Research* and the *Cochrane Database of Systematic Reviews* stand out for having the highest average citations per article, at 57.17 and 70.64, respectively, reflecting the significant influence of their publications. Additionally, the *Cochrane Database of Systematic Reviews* boasts the highest IF, at 8.8, further emphasizing its prominence and influence within this research domain.

**Table 5:** Top 10 journals with the most articles published on vitamin D research.

Ranks	Journal	Record count	Impact factor	H-index	Without self-citations	Average per item
1	Nutrients	50	4.8	17	1060	21.48
2	Osteoporosis International	38	4.2	22	2063	54.42
3	Journal of Bone and Mineral Research	24	5.1	17	1369	57.17
4	Cochrane Database of Systematic Reviews	22	8.8	14	1547	70.64
5	Journal of Bone and Mineral Metabolism	20	2.4	11	452	22.6
6	Medicine (Baltimore)	20	1.3	8	257	12.85
7	Rheumatology International	20	3.2	12	606	31.05
8	PLoS One	19	2.9	13	511	26.95
9	BMC Musculoskeletal Disorders	18	2.2	8	217	12.06
10	International Journal of Molecular Sciences	17	4.9	7	305	18.06

### 3.6. Analysis of research areas

**Figure 7.**
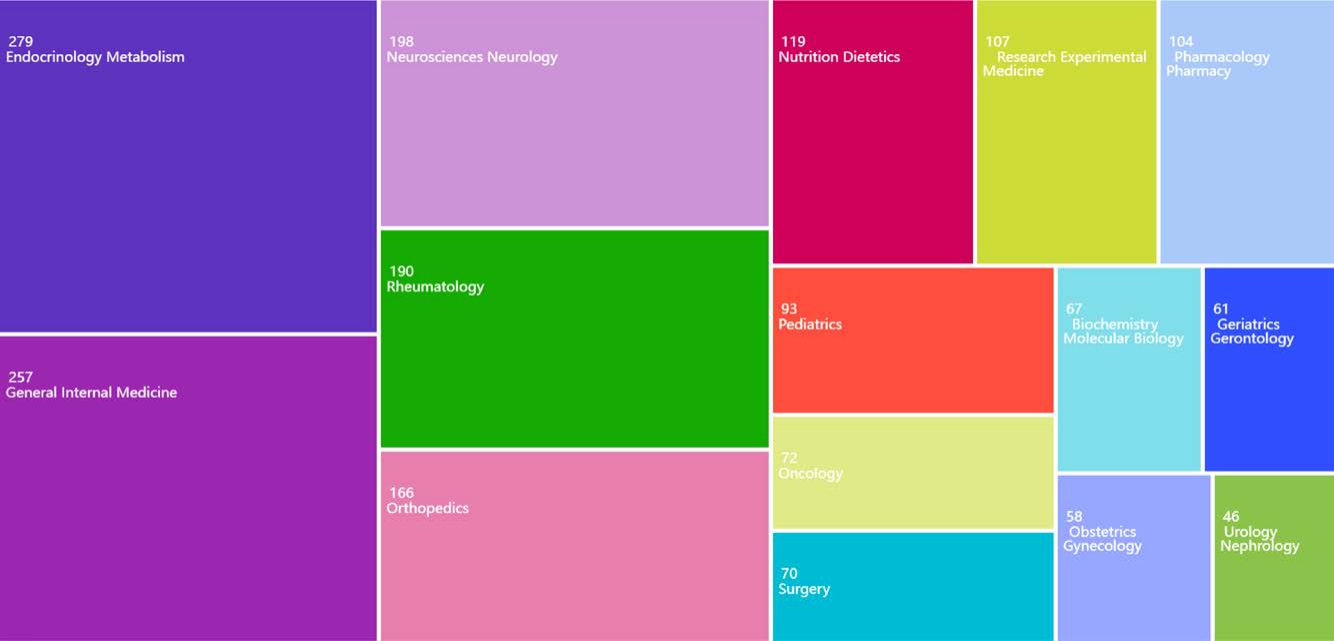
Treemap visualization of research categories. Each rectangle represents a specific research field, with the size of the rectangle corresponding to the number of publications in that category.

Figure 7 illustrates the distribution of the 1899 articles from the WoS database across various research areas, depicted through a rectangular tree diagram.

The keyword combinations used in this analysis suggest a research focus on the role of vitamin D in pain management and fibromyalgia treatment. The largest concentration of articles (279) is found in the field of *Endocrinology & Metabolism*, indicating a strong interest in the biochemical pathways of vitamin D and its potential therapeutic effects on pain and fibromyalgia. The field of *General Internal Medicine* follows closely with 257 articles, where studies likely investigate the broader clinical efficacy of vitamin D supplementation in managing chronic conditions such as fibromyalgia. In *Neurosciences & Neurology*, 198 articles highlight research on the effects of vitamin D on the nervous system, particularly its influence on pain perception and conditions such as neuralgia.

### 3.7. Keyword analysis of research hotspots

**Figure 8.**
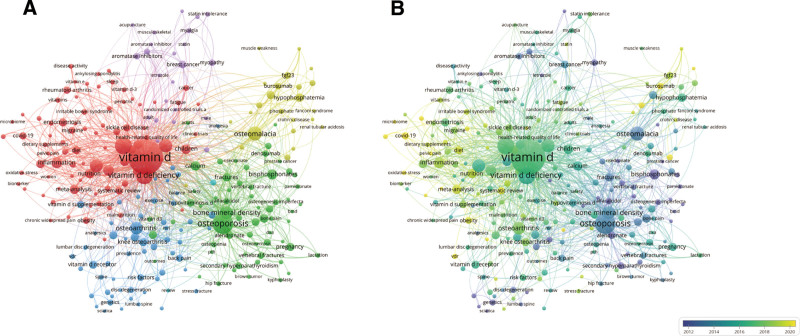
Keyword co-occurrence network analysis related to vitamin D research. (A) The co-occurrence network shows the most frequently appearing keywords in the research field, with the size of each node representing the frequency of keyword appearance, and the thickness of the edges indicating the co-occurrence strength between keywords. (B) The same co-occurrence network is color-coded by the average publication year, where blue nodes represent older studies and yellow nodes represent more recent research.

**Figure 9.**
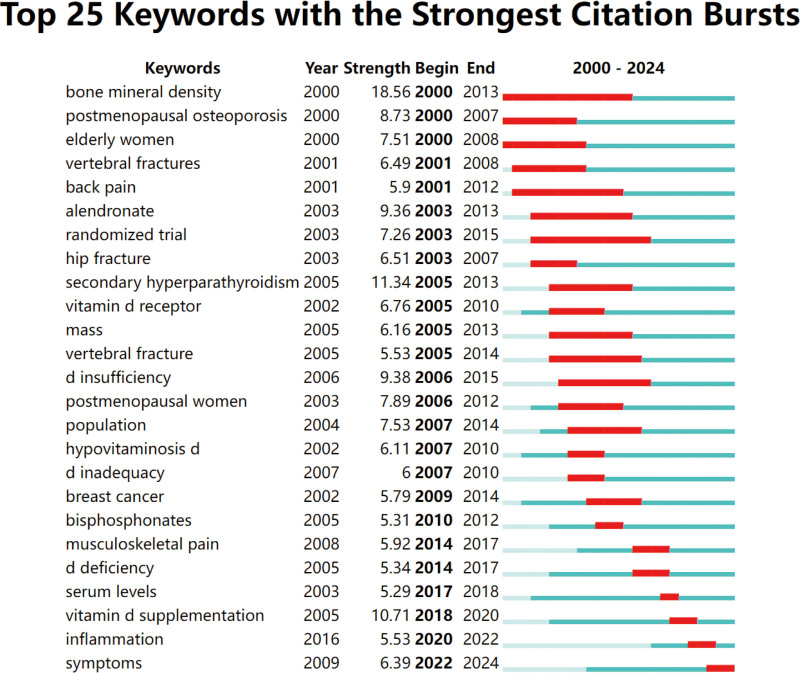
Top 25 keywords with the strongest citation bursts in vitamin D-related research from 2000 to 2024. The figure shows keywords that experienced a sudden increase in citations (citation bursts) over specified time periods. The strength of the burst is depicted numerically, with the beginning and end years of the burst indicated. Red bars represent the period during which the citation burst occurred, while green bars extend beyond the citation burst, indicating ongoing relevance.

We employed VOSviewer to analyze the distribution of author keywords. To enhance the accuracy of cluster analysis and visualization, synonyms were merged, and the minimum occurrence threshold for keywords was set at 15. The resulting clusters are color-coded, with each color representing distinct themes within vitamin D research. As illustrated in Figure 8, the red cluster is concentrated around keywords such as vitamin D, inflammation, nutrition, vitamin D deficiency, and dietary supplements. Research in this cluster emphasizes vitamin D’s role in immune function, particularly its impact on inflammation control and autoimmune diseases. The green cluster, which includes keywords such as osteoporosis, bone mineral density, fractures, osteomalacia, and calcium, focuses on vitamin D’s contribution to bone health, including the prevention and treatment of osteoporosis and other bone-related conditions. The blue cluster features keywords such as VDR, genetics, lumbar spine, sciatica, and risk factors. This cluster explores genetic variations in the VDR and their association with diseases such as sciatica, as well as other risk factors for pain-related conditions. The yellow cluster includes terms such as Coronavirus Disease 2019 (COVID-19), rheumatoid arthritis, endometriosis, migraine, and irritable bowel syndrome. Research in this cluster examines the relationship between vitamin D and a variety of specific health conditions. Finally, the purple cluster is characterized by keywords such as statin intolerance, breast cancer, aromatase inhibitors, muscle weakness, and myalgia, with a focus on drug interactions and treatment-related issues, particularly in the context of vitamin D supplementation. Figure 9 presents the top 25 keyword citation bursts from 2000 to 2024, highlighting periods of rapid growth in specific research topics. The intensity and duration of these bursts provide insights into shifting research priorities. Early research (2000–2010) focused predominantly on terms such as bone mineral density, postmenopausal osteoporosis, and back pain, reflecting an emphasis on bone health, aging populations, and pain management. During the midterm period (2005–2015), keywords such as VDR, insufficiency, randomized trial, and secondary hyperparathyroidism became prominent, indicating a shift toward clinical trials and research on specific diseases within the fields of endocrinology and metabolism. More recent research (2015–2024) has centered around terms such as inflammation and serum levels, reflecting a growing focus on vitamin D supplementation, inflammatory processes, and serum biomarkers.

### 3.8. Synthesis of bibliometric findings

Synthesizing the above results, the bibliometric evidence indicates that research on vitamin D and chronic or idiopathic pain has progressed from early observational and bone-related studies toward a more diverse and interdisciplinary field. Publication growth accelerated markedly after 2010, accompanied by increasing international collaboration and citation impact. Core research clusters consistently centered on inflammation, bone metabolism, and VDR–related mechanisms, while more recent keyword bursts highlight growing interest in supplementation strategies, inflammatory pathways, and serum biomarkers. Despite this expansion, the distribution of research output remains uneven across regions, and interventional studies remain relatively limited compared with observational and mechanistic research, suggesting an imbalance between hypothesis generation and causal validation.

## 4. Discussion

### 4.1. Summary of principal findings

This bibliometric and visualization analysis provides the first macroscopic overview of the research landscape on vitamin D and chronic or idiopathic pain from 2000 to 2023. A total of 1899 publications were identified, showing a marked rise in annual output from only 14 in 2000 to a peak of 184 in 2021. This pattern reflects a steadily increasing scholarly interest in the role of vitamin D within pain-related research. Geographically, the United States, Italy, and the United Kingdom contributed most of the publications, indicating the central role of high-income countries in shaping the discourse. Leading institutions such as Harvard University and the University of Sydney emerged as hubs in the collaborative network, while prolific authors and journals further consolidated the intellectual base. The co-citation and keyword analyses highlighted recurring themes such as inflammation, bone metabolism, and VDR genetics, as well as more recent attention to supplementation trials and evidence synthesis. Together, these findings depict a research field that has grown rapidly, with established clusters of knowledge and emerging frontiers, but also with persistent gaps between observational associations and interventional evidence.

### 4.2. Interpretation of trends in the context of existing knowledge

The dual trajectory of the literature is particularly noteworthy. On one side lies a robust body of observational and mechanistic studies. These works consistently link low serum 25(OH)D with chronic musculoskeletal pain,^[37]^ fibromyalgia,^[38]^ back pain,^[39,40]^ and other pain syndromes, and they explore mechanisms ranging from neuroimmune modulation to musculoskeletal integrity and central sensitization. On the other side lies the interventional literature, primarily RCTs and systematic reviews.^[41,42]^ These studies test whether correcting vitamin D deficiency alleviates pain symptoms, but results have been highly inconsistent. Some trials have reported reductions in pain scores in specific subgroups, such as individuals with low baseline 25(OH)D, whereas others report no clinically significant benefit. Several recent meta-analyses have echoed this heterogeneity, concluding that supplementation cannot yet be recommended as a universal analgesic strategy. This divergence between “association-rich” and “intervention-poor” evidence is a hallmark of the field, and bibliometric mapping helps clarify how it arose: the numerical dominance of observational and mechanistic studies created a strong signal of association, but relatively few high-quality RCTs exist to test causality.

### 4.3. Broader scientific and clinical context

The observed growth in publications parallels broader developments in both pain medicine and nutritional science. Chronic pain represents one of the most significant global health burdens, affecting quality of life, mental health, and socioeconomic outcomes.^[43]^ During the same period, vitamin D gained attention beyond its traditional role in bone metabolism,^[44,45]^ with emerging links to immune regulation,^[46]^ cardiovascular health,^[47,48]^ cancer risk,^[49]^ and neuropsychiatric disorders.^[50]^ The intersection of these 2 trajectories – chronic pain as a pressing clinical problem and vitamin D as a pleiotropic micronutrient – naturally stimulated research interest. Public health campaigns around vitamin D deficiency and widespread testing of serum 25(OH)D further reinforced its visibility. The bibliometric trends, therefore, reflect not only scientific curiosity but also societal and clinical drivers.

### 4.4. Clinical heterogeneity and research controversies

From a clinical standpoint, the implications are complex.^[51]^ On the one hand, epidemiological evidence strongly suggests that vitamin D deficiency is more common among individuals with chronic pain, and mechanistic studies provide plausible pathways for how deficiency may exacerbate pain.^[52]^ On the other hand, the interventional literature shows that supplementation does not consistently reduce pain across populations. The heterogeneity arises from multiple factors: variability in baseline vitamin D status, differences in supplementation regimens and formulations, inconsistent definitions of pain outcomes, and diverse pain phenotypes ranging from musculoskeletal to neuropathic to idiopathic conditions.^[53]^ For example, trials in knee osteoarthritis and nonspecific low back pain have reported mixed outcomes, while studies in fibromyalgia often show modest benefits only in vitamin D–deficient subgroups.^[54,55]^ This suggests that vitamin D may act less as a universal analgesic and more as a modifier of pain in specific biological or clinical contexts.^[51]^ Clinicians should therefore avoid indiscriminate supplementation for pain relief, but screening for and correcting deficiencies remains justified for musculoskeletal health and may confer ancillary benefits for selected pain patients.^[52,56]^

### 4.5. Research gaps and future priorities

The bibliometric evidence points toward several pressing gaps that require strategic attention. A first priority is the establishment of causality. While mechanistic hypotheses abound, robust longitudinal studies that track vitamin D status, inflammatory markers, genetic variation in the VDR, and pain trajectories are scarce.^[52]^ Such integrative designs could help clarify whether vitamin D deficiency is a driver of pain, a consequence of reduced outdoor activity in patients with pain, or both. Second, clinical trials need to be larger, longer, and more precisely stratified. Stratification by baseline 25(OH)D is particularly critical, as supplementation may only be effective in deficient individuals, yet many trials have enrolled heterogeneous populations without prespecified subgroup analyses.^[57]^ Similarly, differentiating between nociceptive, neuropathic, and idiopathic pain syndromes will allow for a more tailored evaluation of therapeutic potential.^[58,59]^

A further research gap lies in the underrepresentation of idiopathic pain syndromes.^[60,61]^ Our mapping shows that conditions such as burning mouth syndrome, vulvodynia, and chronic pelvic pain remain largely absent from the vitamin D literature, despite their disabling impact and poorly understood etiology.^[62–64]^ Addressing these “cold spots” will require targeted funding and multidisciplinary collaboration among pain specialists, endocrinologists, and nutrition scientists.^[65,66]^ Beyond disease categories, nutritional and systems biology perspectives warrant more attention.^[67,68]^ Rather than examining vitamin D in isolation, future studies should consider interactions with other nutrients such as magnesium and vitamin K, as well as diet-based or lifestyle interventions.^[69,70]^ Finally, the geographic concentration of research in high-income countries leaves significant gaps in global understanding.^[71,72]^ Chronic pain prevalence, vitamin D deficiency rates, sunlight exposure, and dietary habits differ substantially across regions, yet low- and middle-income countries are underrepresented. International multicenter consortia could not only improve generalizability but also uncover population-specific moderators.

### 4.6. Strengths and limitations of the present bibliometric study

This study offers a number of strengths. By analyzing more than 2 decades of publications, we provide a longitudinal view of how the field has evolved, from early exploratory associations to emerging interventional research.^[35,73]^ The use of multiple bibliometric tools, including VOSviewer and CiteSpace, enabled complementary perspectives on structural patterns (e.g., co-citation clusters, collaboration networks) and temporal dynamics (e.g., keyword bursts, thematic evolution).^[74]^ Such a dual perspective helps capture both the intellectual foundations of the field and its research frontiers, providing strategic intelligence for researchers, funders, and clinicians.^[75]^

Nevertheless, the limitations inherent to bibliometric methods must be acknowledged. Our reliance on the WoSCC and English-language publications introduces selection bias and may underestimate contributions from non-English or regional journals.^[76]^ Bibliometric indicators such as citation counts, IFs, and H-index measure scholarly attention but not necessarily methodological quality or clinical applicability. They are influenced by visibility, language, and cumulative advantage effects, meaning that highly cited work may not always reflect the best evidence. In addition, bibliometrics cannot substitute for systematic reviews and meta-analyses in evaluating efficacy. Observational, mechanistic, and interventional studies differ fundamentally in evidentiary weight, and their aggregation through bibliometric analysis is descriptive rather than evaluative.^[77,78]^ Despite these caveats, the method remains valuable for mapping intellectual structures, identifying underexplored areas, and informing future research priorities.

## 5. Conclusion

This bibliometric analysis systematically reviews global research on vitamin D in relation to chronic or idiopathic pain from 2000 to 2023. The data show that over the past 2 decades, publication output in this field has steadily increased. However, the majority of contributions still originate from high-income countries and are led by a small number of core institutions, prolific authors, and authoritative journals. Additionally, international collaborative networks have played a crucial role in advancing this field.

In terms of research themes, the academic community has long focused on mechanisms related to inflammation, bone metabolism, and the VDR. More recently, there has been a shift toward emerging directions such as supplementation strategies and inflammatory biomarkers. It is noteworthy that the current literature exhibits a significant structural imbalance: while observational studies and mechanistic discussions are abundant, high-quality interventional studies remain relatively scarce.

This study does not aim to assess clinical efficacy or evidence quality but rather seeks to outline the academic landscape and evolutionary trajectory of the field. By revealing existing research gaps, we hope to provide a macro-level reference for future hypothesis-driven research, systematic reviews, and multinational collaborations. Future research should address this gap through well-designed, adequately powered studies and greater international collaboration, particularly in underrepresented regions and idiopathic pain conditions. Overall, bibliometric analysis serves as a valuable tool for understanding the structure, evolution, and research priorities of this field, and for informing the design of future systematic reviews and hypothesis-driven investigations.

## Author contributions

**Conceptualization:** Zhaohui Jiang, Zhou Lin.

**Funding acquisition:** Zhou Lin.

**Methodology:** Yijie Wang, Wei Zhang.

**Data curation:** Zhaohui Jiang, Zhou Lin.

**Project administration:** Zhou Lin.

**Visualization:** Zhaohui Jiang, Zhenzhen Hu, Fang Lin, Zhou Lin.

**Resources:** Wei Zhang, Zhenzhen Hu.

**Software:** Wei Zhang, Zhenzhen Hu.

**Investigation:** Zhaohui Jiang, Yijie Wang.

**Validation:** Zhaohui Jiang, Fang Lin.

**Writing – original draft:** Zhaohui Jiang, Zhenzhen Hu.

**Writing – review & editing:** Zhaohui Jiang, Zhenzhen Hu.
